# Integrated Polarimetric Spectral Imaging Sensor Combining Spectral Imaging and Polarization Modulation Techniques

**DOI:** 10.3390/s26010144

**Published:** 2025-12-25

**Authors:** Zihao Liu, Zhiping Song, Zhengqiang Li, Li Li

**Affiliations:** 1 School of Physics and Optoelectronic Engineering, Anhui University, Hefei 230601, China; b23301079@stu.ahu.edu.cn; 2 Aerospace Information Research Institute, Chinese Academy of Sciences, Beijing 101408, China; lizq@irsa.ac.cn (Z.L.); ll@aircas.ac.cn (L.L.)

**Keywords:** polarimetric spectral imaging, push-broom imaging spectrometer, polarization modulator, data reconstruction, outdoor experiments

## Abstract

**Highlights:**

**What are the main findings?**

- The proposed integration of polarization modulation with push-broom scanning reduces the system volume by approximately 70% and achieves a total weight of around 1.5 kg, compared with traditional technology.
- The developed Fourier transform-based demodulation algorithm achieves accurate reconstruction of polarimetric spectral image information, with a linear polarization measurement error of less than 2.87% verified by experiments.

**What are the implications of the main findings?**

- Retrieval of Earth’s atmospheric composition and optical parameters
- The identification of sensitive bands reduces the number of redundant experiments and guides polarization-based material discrimination.

**Abstract:**

Polarimetric spectral imaging systems have unique application advantages in environmental remote sensing, military target recognition, astronomy, medicine, etc., because of their ability to acquire multidimensional information. However, traditional systems are constrained by complex structures and low spectral resolution, making them unlikely to achieve their full potential. This study proposes a novel polarimetric spectral imaging method for information acquisition to address these shortcomings. The method integrates a polarization modulator (composed of two retarders and one polarizer) into the incident optical path of a push-broom imaging spectrometer for hardware integration. The modulator statically encodes the full polarization spectral information of the measured light into output power spectra, which the spectrometer records as raw spectral image data. Target polarimetric spectral imaging information is then reconstructed from the raw data to realize sensor functions. The system structure, data reconstruction principles, laboratory experiments with typical polarized light sources, and preliminary outdoor experiments verified the system’s correctness and reliability. The results facilitate further expansion of the application scope of polarimetric spectral imaging systems.

## 1. Introduction

As a key vector characteristic of electromagnetic waves, light polarization—combined with intensity and frequency—forms the basis of multidimensional optical sensing [1]. Polarimetric spectral imaging sensors can synchronously capture multidimensional information, including the spectral, polarization, and imaging data of optical radiation (reflection) signals from targets. By fusing data across different dimensions, these sensors overcome the limitation of “same spectrum, same color” in traditional optical imaging, thus demonstrating broad application prospects in fields such as environmental remote sensing, military reconnaissance, astronomical observation, and medical diagnosis. For instance, in environmental remote sensing, they can distinguish between the health status of vegetation and soil types using polarization characteristics [2]; in military reconnaissance, they can penetrate smoke or camouflage coatings to identify targets [3]; and in the medical field, they can assist in the early detection of tumors by leveraging the polarization properties of biological tissues [4].

Existing polarimetric spectral imaging acquisition technologies are primarily categorized into three types, each differing considerably in the methods of acquiring spectral, polarization, and imaging information, with respective advantages and disadvantages in key performance indicators.

Time-Division Measurement Technology: This technology captures spectral images of a target at multiple polarization angles in different time sequences by mechanically rotating polarizers or retarders and then retrieves complete polarization information using the Stokes vector algorithm [5,6,7,8,9]. Spectral information is obtained through the conventional band decomposition of an imaging spectrometer, while imaging information is formed by stitching images acquired frame by frame. Its advantages include a simple optical structure, high polarization accuracy (which relies on high-precision rotating mechanisms), and spectral resolution limited only by the spectrometer itself [10,11]. However, it suffers from low temporal resolution (requiring multiframe acquisition), which easily leads to interframe misalignment in dynamic scenes. Additionally, mechanical moving parts increase the system volume and maintenance costs [12,13,14,15,16].

Channel-Division Measurement Technology: This technology uses beam-splitting prisms or polarizing beam splitters to divide incident light into multiple parallel optical channels. Each channel is equipped with a fixed polarization element and a spectral detection module to synchronously acquire spectral images under different polarization states, and imaging information is synthesized through multichannel image registration [17,18,19,20]. The core advantages of this technology are high temporal resolution (completing multipolarization state acquisition with a single exposure) and strong system stability because of the absence of mechanical moving parts [21,22]. Nevertheless, multichannel optical elements tend to cause inconsistent spectral responses, which require complex calibration algorithms for correction [23]. Moreover, the design with multiple lenses/sensors increases the system volume and cost, and the allocation of pixel resources results in reduced spatial resolution [24].

Division-of-Focal-Plane (DOFP) Measurement Technology: This technology integrates a micropolarizer array (MPA) on the focal plane of an imaging sensor through nanofabrication processes. Adjacent pixels are grouped into a polarization unit (corresponding to different polarization directions). Spectral information is extracted by an integrated microspectral module or subsequent data processing, and imaging information is directly captured by pixel-level polarization units [25,26,27]. Its prominent advantages include a compact system structure (integrated into a single sensor) and high temporal resolution (enabling real-time synchronous acquisition), making it suitable for portable or airborne platforms [28,29]. However, owing to the pixel multiplexing design of the micropolarizer array, the spatial resolution is reduced by 75% (a 2 × 2 pixel unit corresponds to one imaging point). Furthermore, the precision limitation of the nanofabrication process leads to a low polarization extinction ratio, resulting in large polarization measurement errors in low-light environments [30,31].

The three aforementioned technologies face bottlenecks in balancing spectral resolution, temporal resolution, spatial resolution, and polarization accuracy, making it difficult for them to meet the application requirements of current high-dynamic scenarios (e.g., high-speed target recognition), high spatial precision (e.g., microscale medical imaging), and miniaturized platforms (e.g., equipment for individual soldiers). For example, time-division technology cannot adapt to dynamic targets, channel-division technology is difficult to miniaturize, and DOFP technology faces a trade-off between spatial resolution and polarization accuracy [32].

To address the performance bottlenecks of existing technologies, this paper proposes a quasistatic polarimetric spectral imaging technology scheme that integrates push-broom spectral imaging and polarization spectral intensity modulation. The core idea is to integrate a modulation module composed of two retarders and a polarizer at the front end of a commercial push-broom imaging spectrometer, and without mechanical moving parts, the spectral components of the target’s Stokes vector are encoded into carrier signals of different frequencies through optical modulation. Then, Fourier transform demodulation is used to separate the polarization spectral information of each component, and combined with the spatial scanning capability of push-broom imaging, the synchronous acquisition of multidimensional information is achieved [3,10]. This technical scheme offers three core advantages. First, for the synchronous acquisition of multidimensional information, it can capture spectral, polarization, and imaging information with a single push-broom scan through optical modulation instead of time-sequential or multichannel division, and the temporal resolution is improved to the second level, enabling adaptation to dynamic scenes [8,10]. Second, in terms of high indicator balance, the spectral resolution (1 nm) is guaranteed by the push-broom spectrometer, the spatial resolution (960 × 960 pixels) has no loss from pixel multiplexing, and the linear polarization measurement error is less than 2.87%, breaking the indicator trade-off of traditional technologies [5,25]. Third, in terms of light weight and portability, the modulation module adopts an integrated design (with a thickness of 55 mm) that can be directly mounted at the front end of a commercial spectrometer, and the overall system weight is only 1.5 kg, which is 70% smaller in volume than channel-division technology, making it suitable for mobile platforms such as field and airborne applications [33].

The remainder of this manuscript is structured as follows. Section 2 addresses the principles of our proposed polarization spectral imaging system, including system composition and data reconstruction. The results of the indoor and outdoor measurement verification experiments and corresponding experimental results are presented in Section 3. Finally, we conclude our work and discuss the results in Section 4. Section 5 specifies the authors’ contributions, funding sources, acknowledgments, and conflict of interest statements.

## 2. Materials and Methods

### 2.1. Principle of System Composition

Polarization represents the vector nature of electromagnetic radiation, where the electric field vector exhibits directional sensitivity and a dominant vibration orientation in directions perpendicular to electromagnetic wave propagation. The polarization state of light can be completely described by the Stokes parameters. All four Stokes parameters, $S_{0}$, $S_{1}$, $S_{2}$ and $S_{3}$, are measurable. The Stokes vector of the light under testing is expressed as follows:

(1) $$\left[\begin{array}{c} S_{0}\\ S_{1}\\ S_{2}\\ S_{3} \end{array}\right]\;or\;\left[S_{0}S_{1}S_{2}S_{3}\right]$$

In Section 1, three types of sensor systems are listed for polarization information acquisition to acquire the full Stokes vector, all of which have obvious drawbacks. To address this problem, in this study, a polarization spectral modulation sensor system that combines a push-broom imaging spectrometer with a modulator to form a polarization spectral intensity modulation imaging system was designed, resulting in the rapid acquisition of polarization information.

To obtain the spectral data of all pixels in the field of view, the spectral modulation analysis of a single pixel must be performed first. Therefore, a modulator must be mechanically compatible with the lens structure of commercial hyperspectral imagers. This requires precise determination of the diameter of the optical components in the modulator and the inner diameter of the mounting cylinder to ensure nondestructive disassembly and assembly between the modulator and the lens. On the other hand, the modulator needs to achieve optical matching with the hyperspectral imager in terms of spectral range, spectral resolution, and quantization depth. Therefore, the thicknesses of the two retarders and the specifications of the polarizer (such as the spectral range and extinction ratio) were carefully selected to maximize the performance of the hyperspectral system. On the basis of the selected AHG-101 portable push-broom hyperspectral imager, the design parameters of the modulator are as follows.

The thicknesses of the two retarders are 2 mm and 4 mm, respectively, ensuring that the frequency components do not overlap during modulation and demodulation.

The polarizer is an Edmund XP42 linear polarizer (Edmund Optics, Barrington, NJ, USA), with a working wavelength range of 400–700 nm and an extinction ratio exceeding 10,000:1.

The relative angle between the fast axes of the two retarders is 45 ± 0.5°.

The alignment error between the transmission axis of the polarizer and the fast axis of the first retarder is less than 0.5°.

**Figure 1:**
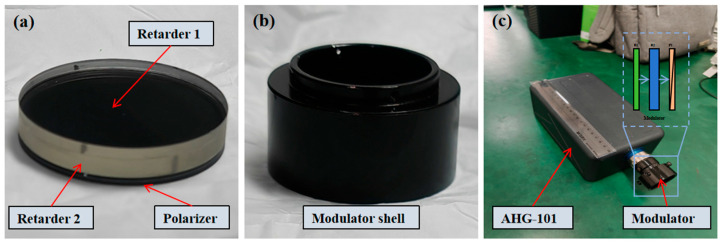
Photos of the components and modulator framework diagram: (**a**) Diagram of the modulator lens assembly; (**b**) modulator shell; (**c**) Diagram of the integrated assembly of the push-broom imaging spectrometer and modulator.

**Figure 2:**
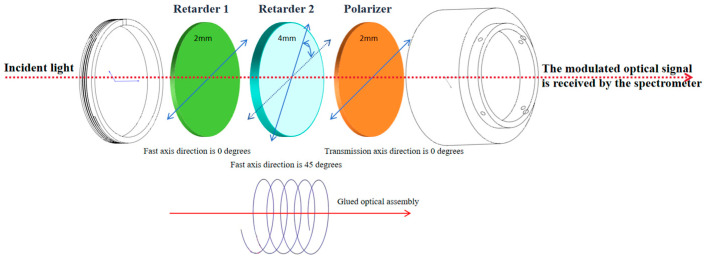
Schematic of the modulator assembly framework. Taking the fast axis of the first 2 mm retarder as the reference, the fast axis direction of the second 4 mm retarder forms a 45° angle with that of the first retarder. Finally, the transmission axis direction of the polarizer is consistent with the fast axis direction of the first retarder.

In accordance with the above specifications, the optical components of the modulator were processed, screened, and cemented and then assembled into a cylindrical housing to form a complete module. Finally, the module was mounted on the front end of the lens of the AHG-101 hyperspectral imager. Photos of the components and the modulator framework diagram are shown in Figure 1 and Figure 2, respectively.

**Figure 3:**
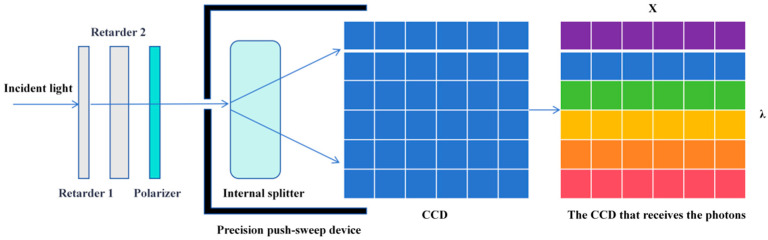
Light-splitting principle of the push-broom imaging spectrometer: a column of spatial data is split into light components and transmitted to the ccd for storage.

The AHG-101 built-in push-broom imaging spectrometer is an instrument that combines an area array CCD detector with a push-broom method to construct a spectral cube by acquiring spectral and spatial information of the target. The optical system decomposes the incident light from the target into different wavelengths and focuses them onto the area array CCD detector. Moreover, the two-dimensional array of the area array CCD detector records the relationship between one spatial dimension and wavelength in a single exposure, and then acquires the second spatial dimension through push-broom scanning over time, thereby obtaining a complete spectral data cube. The horizontal direction (X) corresponds to the spatial resolution of the target (spatial dimension), and the vertical direction (λ) corresponds to the wavelength resolution after light splitting (spectral dimension), as shown in Figure 3.

**Figure 4:**
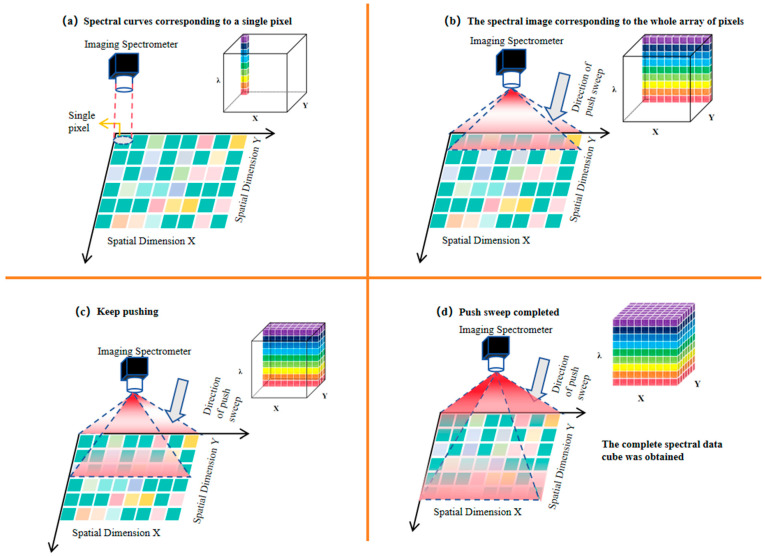
Steps for the imaging spectrometer to acquire the complete data cube within the field of view via push-broom scanning.

After the polarization spectral data of a single pixel or a single row of pixels are acquired, the built-in push-broom module of the AHG-101 spectrometer then initiates a linear scanning process along the spatial direction perpendicular to the pixel row. During this scanning, the spectrometer sequentially collects the modulated spectral data of each subsequent pixel row in the target’s field of view. By stitching together the demodulated and reconstructed data of all the pixel rows obtained through the push-broom scan, a comprehensive three-dimensional spectral data cube was finally formed. In this data cube, the three dimensions correspond to the target’s spatial X-axis, spatial Y-axis (jointly reflecting the two-dimensional spatial distribution), and spectral wavelength (λ-axis, covering the 400–700 nm visible range with a 1 nm spectral resolution), as shown in Figure 4.

### 2.2. Principle of Information Reconstruction

The modulation and superposition of the spectral components of the Stokes vector for the light under testing serve as critical prerequisites for intensity modulation systems to obtain the complete Stokes vector spectral components of a target via a single measurement. This modulation and superposition process is executed by the modulator within the intensity modulation system. The two retarders in the modulator generate the carrier signals required to modulate different incident Stokes vector spectral components, while the polarizer linearly superposes each modulated signal onto the intensity spectrum of its output. The modulation mechanism of the modulator can be derived from the relationship among the Stokes vector spectral components of the light under testing, the cascaded Mueller matrix of the modulator, and the Stokes vector spectral components of the modulator’s output light as follows.

Assuming that the Stokes vector spectral components of the light output from the modulator are denoted as $S^{\prime }(\sigma )$, then

(2) $$S^{\prime }\left(\sigma \right)=\left[\begin{array}{c} S_{0}^{\prime }\left(\sigma \right)\\ S_{1}^{\prime }\left(\sigma \right)\\ S_{2}^{\prime }\left(\sigma \right)\\ S_{3}^{\prime }\left(\sigma \right) \end{array}\right]=M_{p}M_{d2}M_{d1}S\left(\sigma \right)$$

In the equation, $S\left(\sigma \right)$ represents the Stokes vector spectral components of the incident light under testing. $M_{d1}$ is the Mueller matrix of the first retarder, whose fast axis is oriented horizontally; $M_{d2}$ is the Mueller matrix of the second retarder, whose fast axis forms a 45° angle with the horizontal direction; $M_{p}$ is the Mueller matrix of the analyzer, whose transmission axis is aligned horizontally; and σ denotes the wavenumber (=1λ). Therefore,

(3) $$M_{d1}=\left[\begin{array}{cccc} 1 & 0 & 0 & 0\\ 0 & 1 & 0 & 0\\ 0 & 0 & cos\phi_{1}\left(\sigma \right) & sin\phi_{1}\left(\sigma \right)\\ 0 & 0 & -sin\phi_{1}\left(\sigma \right) & cos\phi_{1}\left(\sigma \right) \end{array}\right]$$

(4) $$M_{d2}=\left[\begin{array}{cccc} 1 & 0 & 0 & 0\\ 0 & cos\phi_{2}\left(\sigma \right) & 0 & -sin\phi_{2}\left(\sigma \right)\\ 0 & 0 & 1 & 0\\ 0 & sin\phi_{2}\left(\sigma \right) & 0 & cos\phi_{2}\left(\sigma \right) \end{array}\right]$$

(5) $$M_{p}=\left[\begin{array}{cccc} 1 & 1 & 0 & 0\\ 1 & 1 & 0 & 0\\ 0 & 0 & 0 & 0\\ 0 & 0 & 0 & 0 \end{array}\right]$$

We assume that the Stokes vector spectral components of the light under testing are

(6) $$s\left(\sigma \right)=\left[\begin{array}{c} S_{0}\left(\sigma \right)\\ S_{1}\left(\sigma \right)\\ S_{2}\left(\sigma \right)\\ S_{3}\left(\sigma \right) \end{array}\right]$$

then

(7) $$\left[\begin{array}{c} S_{0}^{\prime }\left(\sigma \right)\\ S_{1}^{\prime }\left(\sigma \right)\\ S_{2}^{\prime }\left(\sigma \right)\\ S_{3}^{\prime }\left(\sigma \right) \end{array}\right]\;\begin{array}{c} =\frac{1}{2}\left[\begin{array}{cccc} 1 & 1 & 0 & 0\\ 1 & 1 & 0 & 0\\ 0 & 0 & 0 & 0\\ 0 & 0 & 0 & 0 \end{array}\right]\left[\begin{array}{cccc} 1 & 0 & 0 & 0\\ 0 & cos\phi_{2}\left(\sigma \right) & 0 & -sin\phi_{2}\left(\sigma \right)\\ 0 & 0 & 1 & 0\\ 0 & sin\phi_{2}\left(\sigma \right) & 0 & cos\phi_{2}\left(\sigma \right) \end{array}\right]\left[\begin{array}{cccc} 1 & 0 & 0 & 0\\ 0 & 1 & 0 & 0\\ 0 & 0 & cos\phi_{1}\left(\sigma \right) & sin\phi_{1}\left(\sigma \right)\\ 0 & 0 & -sin\phi_{1}\left(\sigma \right) & cos\phi_{1}\left(\sigma \right) \end{array}\right]\left[\begin{array}{c} S_{0}\left(\sigma \right)\\ S_{1}\left(\sigma \right)\\ S_{2}\left(\sigma \right)\\ S_{3}\left(\sigma \right) \end{array}\right]\\ =\frac{1}{2}[\begin{array}{c} S_{0}\left(\sigma \right)+S_{1}\left(\sigma \right)cos\phi_{2}\left(\sigma \right)+S_{2}\left(\sigma \right)sin\phi_{1}\left(\sigma \right)sin\phi_{2}\left(\sigma \right)-S_{3}\left(\sigma \right)cos\phi_{1}\left(\sigma \right)sin\phi_{2}\left(\sigma \right)\\ S_{0}\left(\sigma \right)+S_{1}\left(\sigma \right)cos\phi_{2}\left(\sigma \right)+S_{2}\left(\sigma \right)sin\phi_{1}\left(\sigma \right)sin\phi_{2}\left(\sigma \right)-S_{3}\left(\sigma \right)cos\phi_{1}\left(\sigma \right)sin\phi_{2}\left(\sigma \right)\\ 0\\ 0 \end{array}] \end{array}$$

The intensity spectrum of the pixel can be expressed as follows after passing through the modulator:

(8) $$P\left(\sigma \right)=\frac{1}{2}S_{0}\left(\sigma \right)+\frac{1}{2}S_{1}\left(\sigma \right)cos\phi_{2}\left(\sigma \right)+\frac{1}{2}S_{2}\left(\sigma \right)sin\phi_{1}\left(\sigma \right)sin\phi_{2}\left(\sigma \right)\;-\frac{1}{2}S_{3}\left(\sigma \right)cos\phi_{1}\left(\sigma \right)sin\phi_{2}\left(\sigma \right)$$

In Equation (8), the phase delay of the j-th retarder (j = 1, 2) is expressed as $\phi_{j}\left(\sigma \right)=2\pi B\left(\sigma \right)D_{j}\sigma$, where $B(\sigma )=n_{e}(\sigma )-n_{o}(\sigma )$ denotes the difference in the refractive index between the extraordinary (e) and ordinary (o) rays in the retarders and $D_{j}$ (j = 1, 2) represents the thickness of the two retarders. For the quartz crystal retarders (Abbe number: 67.8) used in this experiment, B(σ) exhibits slight dispersion (i.e., variation with wavenumber σ) within the system’s working spectral range (400–700 nm), but the variation is sufficiently small to be negligible. To simplify the principle analysis while ensuring calculation accuracy, B(σ) can be approximated as a constant in the working band. Thus, $\phi_{j}\left(\sigma \right)$ shows a linear proportional relationship with σ.

A detailed breakdown of Equation (8) further clarifies the signal modulation mechanism. By introducing a complex vector to consolidate the second and third Stokes parameters:

(9) $$S_{23}\left(\sigma \right)=S_{2}\left(\sigma \right)+iS_{3}\left(\sigma \right)$$

$S_{2}\left(\sigma \right)$ and $S_{3}\left(\sigma \right)$ can be rewritten using the modulus and argument of:

(10) $$S_{2}\left(\sigma \right)=|S_{23}\left(\sigma \right)|cos(arg\left\{S_{23}\left(\sigma \right)\right\})$$

(11) $$S_{3}\left(\sigma \right)=|S_{23}\left(\sigma \right)|sin(arg\left\{S_{23}\left(\sigma \right)\right\})$$

Substituting Equations (9)–(11) into Equation (8) and simplifying via trigonometric identities (including product-to-sum formulas) yields:

(12) $$\begin{array}{ll} P\left(\sigma \right) & =\frac{1}{2}S_{0}\left(\sigma \right)+\frac{1}{2}S_{1}\left(\sigma \right)cos\left[2\pi L_{2}\sigma \right]+\frac{1}{2}\left|S_{23}\left(\sigma \right)\right|cos\left[arg\left\{S_{23}\left(\sigma \right)\right\}\right]sin\phi_{1}\left(\sigma \right)sin\phi_{2}\left(\sigma \right)\\ & -\frac{1}{2}\left|S_{23}\left(\sigma \right)\right|sin\left[arg\left\{S_{23}\left(\sigma \right)\right\}\right]cos\phi_{1}\left(\sigma \right)sin\phi_{2}\left(\sigma \right)\\ & =\frac{1}{2}S_{0}\left(\sigma \right)+\frac{1}{2}S_{1}\left(\sigma \right)cos\left[2\pi L_{2}\sigma \right]+\frac{1}{2}\left|S_{23}\left(\sigma \right)\right|sin\phi_{2}\left(\sigma \right)\left\{\begin{array}{c} cos\left[arg\left\{S_{23}\left(\sigma \right)\right\}\right]sin\phi_{1}\left(\sigma \right)\\ -sin\left[arg\left\{S_{23}\left(\sigma \right)\right\}\right]cos\phi_{1}\left(\sigma \right) \end{array}\right\}\\ & =\frac{1}{2}S_{0}\left(\sigma \right)+\frac{1}{2}S_{1}\left(\sigma \right)cos\left[2\pi L_{2}\sigma \right]+\frac{1}{2}\left|S_{23}\left(\sigma \right)\right|sin\phi_{2}\left(\sigma \right)sin\left[\phi_{1}\left(\sigma \right)-arg\left\{S_{23}\left(\sigma \right)\right\}\right]\\ & =\frac{1}{2}S_{0}\left(\sigma \right)+\frac{1}{2}S_{1}\left(\sigma \right)cos\left[2\pi L_{2}\sigma \right]+\frac{1}{4}\left|S_{23}\left(\sigma \right)\right|\left\{\begin{array}{c} cos\left[\phi_{2}\left(\sigma \right)-\phi_{1}\left(\sigma \right)+arg\left\{S_{23}\left(\sigma \right)\right\}\right]\\ -cos\left[\phi_{2}\left(\sigma \right)+\phi_{1}\left(\sigma \right)-arg\left\{S_{23}\left(\sigma \right)\right\}\right] \end{array}\right\}\\ & =\frac{1}{2}S_{0}\left(\sigma \right)+\frac{1}{2}S_{1}\left(\sigma \right)cos\left[2\pi L_{2}\sigma \right]+\frac{1}{4}\left|S_{23}\left(\sigma \right)\right|cos\left[2\pi \left(L_{2}-L_{1}\right)\sigma +arg\left\{S_{23}\left(\sigma \right)\right\}\right]\\ & -\frac{1}{4}\left|S_{23}\left(\sigma \right)\right|cos\left[2\pi \left(L_{2}+L_{1}\right)\sigma -arg\left\{S_{23}\left(\sigma \right)\right\}\right] \end{array}$$

In the equations, $L_{j}$ (where j=1, 2) is defined as $L_{j}=B(\sigma )D_{j}$, which represents the optical path difference between the ordinary ray (o-ray) and extraordinary ray (e-ray) when the measured light passes through the retarder. The cosine terms in Equation (15) correspond to distinct carrier frequencies determined by $L_{1}$ and $L_{2}$. Carrier frequency of $cos\left[2\pi L_{2}\sigma \right]$, $cos\left[2\pi \left(L_{2}-L_{1}\right)\sigma +arg\left\{S_{23}\left(\sigma \right)\right\}\right]$ and $cos\left[2\pi \left(L_{2}+L_{1}\right)\sigma -arg\left\{S_{23}\left(\sigma \right)\right\}\right]$:

(13) $$f_{2}=L_{2}=B(\sigma )D_{2}$$

(14) $$f_{2-1}=L_{2}-L_{1}=B\left(\sigma \right){(D}_{2}-D_{1})$$

(15) $$f_{2+1}=L_{2}+L_{1}=B\left(\sigma \right){|D}_{2}+D_{1}|$$

By rationally designing the thicknesses of the two retarders (2 mm and 4 mm in this study), the three carrier frequencies corresponding to the sinusoidal terms-specifically $f_{2}=4B\left(\sigma \right),\;f_{2+1}=6B\left(\sigma \right),\;and\;\;f_{2-1}=2B\left(\sigma \right)$ can be separated from each other after Fourier transform of the modulated spectral data, which effectively avoids frequency overlap during subsequent demodulation processes. Through theoretical calculations and pre-experiments, we designed the retarders with thickness of 2 mm and 4 mm: it not only enables clear separation of signals in the optical path difference domain but also avoids adverse effects caused by excessively thick retarders—such as increased optical absorption loss, prolonged light propagation time leading to signal phase distortion, and added structural bulk that compromises the modulator’s compactness and operational stability. The retarders are then fixed in the modulator assembly, ensuring reliable system structure stability during actual operation.

Through subsequent filtering and demodulation, the spectral components of the four Stokes parameters can be accurately recovered, thus completing the acquisition of the polarization spectral information for a single pixel. According to the properties of the inverse Fourier transform, the general form of the autocorrelation function obtained after Fourier transforming Equation (12) is shown in Equation (16).

(16) $$\begin{array}{c} C\left(h\right)=A_{0}\left(h\right)+A_{1}\left(h-\{L_{2}-L_{1}\}\right)+A_{1}^{*}\left(-h-\{L_{2}-L_{1}\}\right)+A_{2}\left(h-L_{2}\right)\\ +A_{2}^{*}\left(-h-L_{2}\right)+A_{3}\left(h-\{L_{2}+L_{1}\}\right)+A_{3}^{*}\left(-h-\{L_{2}+L_{1}\}\right) \end{array}$$

Each term in Equation (16) is the result of transforming Equation (12) from the wavenumber domain to the optical path difference domain, where:

(17) $$A_{0}\left(h\right)=F^{-1}\left\{\frac{1}{2}S_{0}\left(\sigma \right)\right\}$$

In Equation (17), the operator $F^{-1}\{\}$ denotes the inverse Fourier transform, and F{} denotes the Fourier transform. Transforming the term containing $S_{1}$ in the result of P(σ) yields Equation (18).

(18) $$\frac{1}{2}S_{1}\left(\sigma \right)cos\left(2\pi L_{2}\sigma \right)=\frac{1}{2}S_{1}\left(\sigma \right)\left(\frac{e^{i2\pi L_{2}\sigma }+e^{-i2\pi L_{2}\sigma }}{2}\right)\;=\frac{1}{4}S_{1}\left(\sigma \right)\left(e^{i2\pi L_{2}\sigma }+e^{-i2\pi L_{2}\sigma }\right)$$

Thus, when letting:

(19) $$A_{2}\left(h\right)=F^{-1}\left\{\frac{1}{4}S_{1}\left(\sigma \right)\right\}$$

It follows that:

(20) $$F^{-1}\left\{\frac{1}{4}S_{1}\left(\sigma \right)e^{i2\pi L_{2}\sigma }\right\}=A_{2}\left(h-L_{2}\right)$$

(21) $$F^{-1}\left\{\frac{1}{4}S_{1}\left(\sigma \right)e^{-i2\pi L_{2}\sigma }\right\}=A_{2}^{*}\left(-h-L_{2}\right)$$

Moreover, the positive term containing $S_{23}$ in the result of P(σ) can be transformed to obtain Equation (22).

(22) $$\begin{array}{c} \frac{1}{4}\left|S_{23}\left(\sigma \right)\right|cos\left[2\pi \left(L_{2}-L_{1}\right)\sigma +arg\left\{S_{23}\left(\sigma \right)\right\}\right]\\ =\frac{1}{4}\left|S_{23}\left(\sigma \right)\right|\left[\frac{e^{i\left[2\pi \left(L_{2}-L_{1}\right)\sigma +arg\left\{S_{23}\left(\sigma \right)\right\}\right]}+e^{-i\left[2\pi \left(L_{2}-L_{1}\right)\sigma +arg\left\{S_{23}\left(\sigma \right)\right\}\right]}}{2}\right]\\ =\frac{1}{8}S_{23}e^{i\left[2\pi \left(L_{2}-L_{1}\right)\sigma \right]}+\frac{1}{8}S_{23}^{*}e^{-i\left[2\pi \left(L_{2}-L_{1}\right)\sigma \right]} \end{array}$$

Herein, let:

(23) $$A_{1}\left(h\right)=F^{-1}\left\{\frac{1}{8}S_{23}\left(\sigma \right)\right\}$$

This yields:

(24) $$F^{-1}\left\{\frac{1}{8}S_{23}\left(\sigma \right)e^{i2\pi \left(L_{2}-L_{1}\right)\sigma }\right\}=A_{1}\left[h-\left(L_{2}-L_{1}\right)\right]$$

(25) $$F^{-1}\left\{\frac{1}{8}S_{2}^{*}\left(\sigma \right)e^{-i2\pi \left(L_{2}-L_{1}\right)\sigma }\right\}=A_{1}^{*}\left[-h-\left(L_{2}-L_{1}\right)\right]$$

By the same logic, the transformed result of the negative term containing $S_{23}$ in the result of P(σ) can also be derived as follows:

(26) $$F^{-1}\left\{-\frac{1}{8}S_{23}\left(\sigma \right)e^{-i\left[2\pi \left(L_{2}+L_{1}\right)\sigma \right]}\right\}=A_{3}\left[h-\left(L_{2}+L_{1}\right)\right]$$

(27) $$F^{-1}\left\{-\frac{1}{8}S_{23}^{*}\left(\sigma \right)e^{i\left[2\pi \left(L_{2}+L_{1}\right)\sigma \right]}\right\}=A_{3}^{*}\left[-h-\left(L_{2}+L_{1}\right)\right]$$

In this way, the general form of the Fourier transform of P(σ) from the wavenumber domain to the optical path difference domain is obtained, as shown in Equation (16) above.

**Figure 5:**
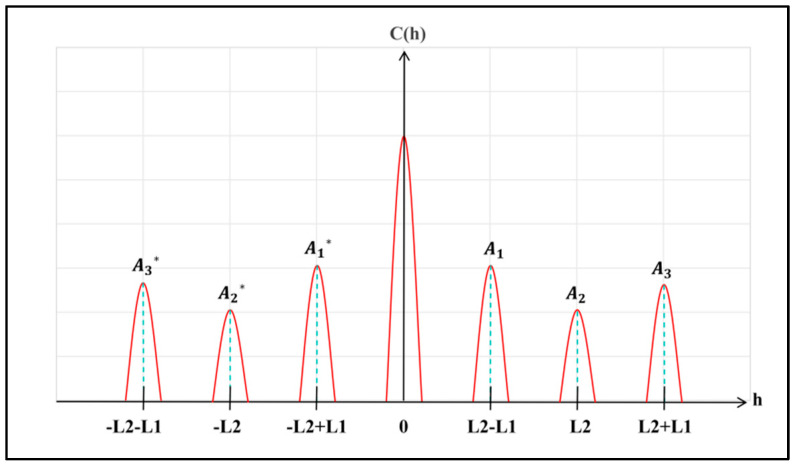
Schematic diagram of theoretical image in autocorrelation domain (optical path difference domain) after modulator intensity modulation and inverse Fourier transform.

Thus, the distribution diagram of the autocorrelation function of P(σ) in the optical path difference system is shown in Figure 5.

From the relationship between the central frequencies of the aforementioned carrier signals and $L_{j}$, when the thickness ratio of the two retarders is set to *D*_2_:*D*_1_ = 2:1, the intervals between the central frequencies become equal. This equidistant distribution ensures clear separation of spectral components in the optical path difference domain after inverse Fourier transform, laying a foundational basis for effective filtering. The absolute values of $D_{1}$ and $D_{2}$ are determined through comprehensive consideration of multiple factors: excessively thick retarders would reduce light transmittance and degrade system signal-to-noise ratio; meanwhile, to maintain the compactness and portability of the modulator, the total thickness of the two retarders must be controlled. Additionally, the maximum allowable thickness of $D_{1}$ is constrained by the formula $D_{1max}=\frac{S_{n}}{\left(\sigma_{max}-\sigma_{min})\times B(\sigma \right)}\times \frac{1}{7}$ (where $S_{n}$ is the number of sampling points of the spectrometer, $\sigma_{max}-\sigma_{min}$ represents the wavenumber range, and B(σ) = $n_{e}\left(\sigma \right)-n_{o}\left(\sigma \right)$ denotes the refractive index difference between extraordinary and ordinary rays). For our system with 720 sampling points, $D_{1max}$ is calculated as 9.3 mm, ensuring no frequency aliasing under the given sampling resolution. By balancing these factors, we selected $D_{1}=2\;\mathrm{mm}$ and $D_{2}=4\;\mathrm{mm}$ (maintaining the 2:1 ratio) as the thicknesses.

The positions of coordinate points such as L2−L1, L2, L2+L1, −L2−L1, −L2, and −L2+L1 in Figure 5 within the optical path difference domain depend entirely on the thicknesses of the two retarders. In this study, retarders with thicknesses of 2 mm and 4 mm are used, which ensures no aliasing occurs between the aforementioned items. Thus, filters can be used to extract the terms corresponding to Equations (17), (20), and (24) from Equation (16). Performing Fourier transform on each extracted term separately yields the following equations:

(28) $$F\left\{A_{0}\left(h\right)\right\}=\frac{1}{2}S_{0}\left(\sigma \right)$$

(29) $$F\left\{A_{2}\left(h-L_{2}\right)\right\}=\frac{1}{4}S_{1}\left(\sigma \right)e^{i2\pi L_{2}\sigma }$$

(30) $$F\left\{A_{1}\left[h-\left(L_{2}-L_{1}\right)\right]\right\}=\frac{1}{8}e^{i2\pi \left(L_{2}-L_{1}\right)\sigma }\left[S_{2}\left(\sigma \right)+iS_{3}\left(\sigma \right)\right]$$

**Figure 6:**
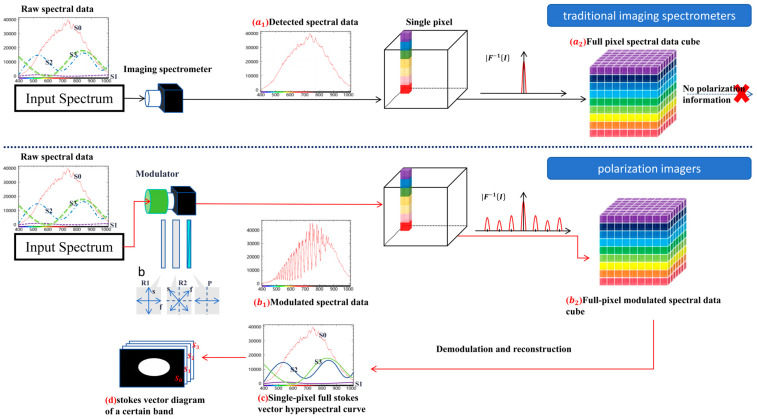
Difference in data cube acquisition between traditional imaging spectrometers and polarization imagers.

Based on Equations (27)–(29), the $S_{0}(\sigma )$, $S_{1}(\sigma )$, $S_{2}(\sigma )$, and $S_{3}(\sigma )$ of the measured light can be calculated. The above steps correspond to the demodulation process for the Stokes vector of a single pixel. By performing a push-broom scan and repeating this process, the demodulation of the Stokes vector for all pixels can be achieved. A comparison diagram between the data cube acquired by a conventional imaging spectrometer and the modulated data cube acquired by the polarimetric imaging spectrometer is shown in Figure 6 below.

Through integration with the push-broom module of the imaging spectrometer, as shown in Figure 6, this process is repeated for all pixels across the target’s scanning swath, allowing the reconstruction of a complete hyperspectral polarization image.

## 3. Results

To validate the working principle of the polarimetric hyperspectral imaging sensor as well as the correctness of its hardware and software design, we conducted a series of indoor and outdoor measurement experiments, followed by data processing and analysis. In the indoor setting, various typical polarized light spots were generated using an integrating sphere and a polarizer, whose light transmission axis direction can be accurately set by mounting on a precision turntable. The correctness of the sensor’s principle and design can be verified by comparing the reconstructed results with theoretical predictions. An outdoor experiment was performed to further verify the practical applicability of the sensor in real-world scenarios, and an indoor validation of its design correctness was performed. Through the polarization imaging spectrometer, a modulated spectral data cube can be obtained. It modulates the polarization information of light into the intensity spectrum P(σ), as shown in Equation (8). This data cube is imported into our demodulation algorithm software. Combined with the previous demodulation principle, the inverse Fourier transform is performed on the raw data to obtain C(h) in the optical path difference domain (Equation (16)). Subsequently, filtering is applied to different frequency components, and finally, these components are converted back to the wavenumber domain via Fourier transform to demodulate the Stokes vector. After the processing software processes the modulated spectra of all pixels according to the above workflow, the polarization spectral image can be reconstructed.

### 3.1. Indoor Validation of the Integrated Sphere Polarization Measurement

**Figure 7:**
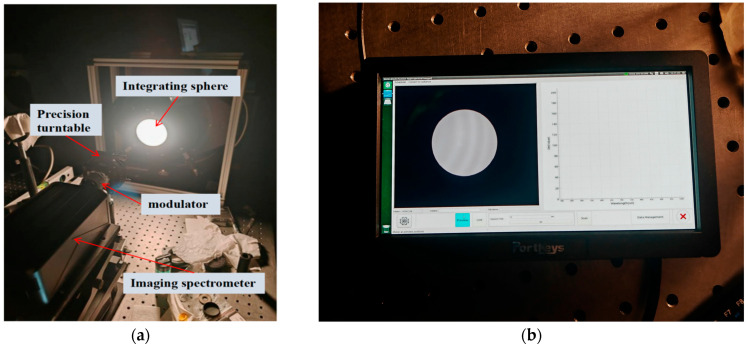
Schematic of the experimental setup of the integrated sphere in a dark room: (**a**) Schematic of the setup assembly; (**b**) The display shows the integrated sphere within the field of view, with the integrated sphere positioned at the center.

To verify the reliability of the system’s polarization measurement, an integrated sphere experiment was conducted indoors. Two common polarization directions were set for the measurement: horizontal and vertical. Measuring representative polarization directions allows for an intuitive evaluation of the system’s polarization stability across different orientations. For the experimental setup, the polarization imaging spectrometer was placed on an optical platform, with a precision optical turntable positioned at the front to adjust the transmission axis direction of the polarizer. Moreover, the light spot of the integrated sphere was vertically illuminated at the center of the imaging system’s field of view, as shown in Figure 7, where the light spot of the integrated sphere could be clearly observed.

The indoor experimental environment and device performance are described as follows.

- A calibration laboratory with walls covered in light-absorbing material was used to minimize stray light interference.
- The Labsphere USLR-A12F-XAN2-P integrated sphere light source offers a spectral output range of 300–2400 nm. After 30 min of preheating with three xenon lamps, it stabilized at 175 W, with fluctuations <0.5%.
- An Edmund linear polarizer (extinction ratio of 10,000:1) was mounted on a THOR LABS PRM1 high-precision rotation stage, featuring a 5 arc-min Vernier resolution and ±7° fine adjustment capability (angular repositioning error ±0.1°) for precise polarization control.
- The AHG-101 hyperspectral imager operated at the following imaging parameters: aperture of f/5.6, integration time of 5 ms, spectral resolution of 1 nm, and spatial resolution of 960 × 960 pixels.

1. Construction, Measurement, and Reconstruction of Horizontally Polarized Light Spots

**Figure 8:**
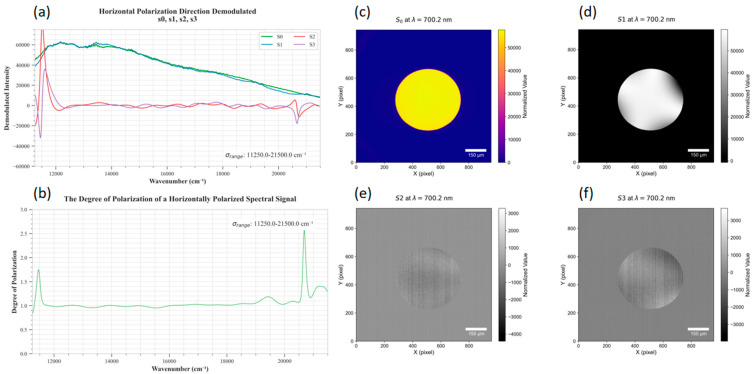
(**a**,**b**) Stokes curves for a single pixel in the horizontal polarization direction; (**c**–**f**): degree of the polarization curves and the filtered full-frame Stokes images $s_{0}$, $s_{1}$, $s_{2}$, and $s_{3}$.

The results of reconstructing the four Stokes parameter spectra of a randomly selected pixel within a horizontally polarized light spot, as well as the full-pixel Stokes images at the center wavelength of 700.2 nm, are shown in Figure 8.

2. Construction, Measurement, and Reconstruction of Vertically Polarized Light Spots

**Figure 9:**
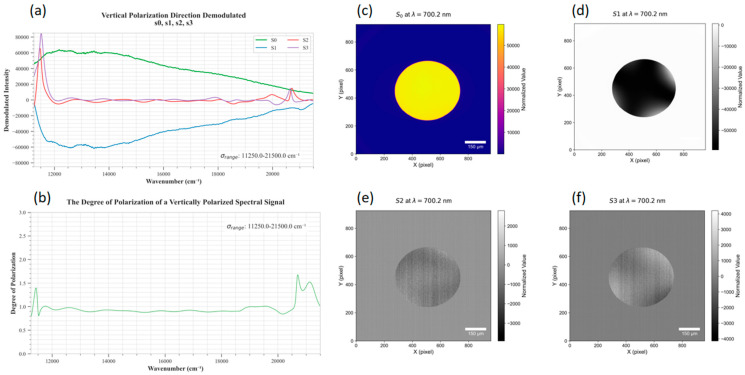
(**a**,**b**) Stokes curves for a single pixel in the vertical polarization direction; (**c**–**f**) degree of the polarization curves and the filtered full-frame Stokes images $s_{0}$, $s_{1}$, $s_{2}$, and $s_{3}$.

The procedure for constructing, measuring, and reconstructing the vertically polarized light spot is the same as that in (1), except that the transmission axis of the polarizer was adjusted to the vertical direction using the precision rotation stage. The results are shown in Figure 9.

3. Analysis Results

As shown in Figure 7, the Stokes spectral components $s_{0}(\lambda )$ and $s_{1}(\lambda )$ of a randomly selected pixel in the horizontally polarized light spot exhibit approximately equal amplitudes and are in phase. In contrast, the corresponding $s_{0}(\lambda )$ and $s_{1}(\lambda )$ components in the vertically polarized light spot are also similar in amplitude but are in the opposite phase. In both cases, the degree of the polarization spectra is close to 1 within the effective measurement wavelength range, which is consistent with the theoretical expectations.

To quantitatively evaluate the reconstruction accuracy, this study selected three different wavelength bands and focused on pixels near the center of the integrating sphere projection. The analysis involved comparing the actual Mueller matrix values with the theoretical values under horizontally and vertically polarized light. Specifically, $S_{0}$ was normalized to 1, and the normalized results of $S_{1}$, $S_{2}$ and $S_{3}$ were calculated. The error analysis results are presented in Table 1. The results show that the normalized value of $S_{1}$ is close to +1 under horizontal polarization and -1 under vertical polarization, with an average error of 2.87%. In both polarization directions, the normalized values of $S_{2}$ and $S_{3}$ are close to 0, with average errors of 2.0% and 1.3%, respectively.

The reconstruction results from the laboratory experiments confirm the correctness of the hardware and software design of the polarimetric hyperspectral imaging sensor. The feasibility and effectiveness of the proposed hyperspectral imaging approach have been validated in practical applications.

In this study, the reconstructed degree of polarization (DoP) exhibits slight exceedances of 1 at the edges of the images. This is primarily attributed to these data lying outside the system’s effective wavelength range (400–700 nm), where deviations in the performance of polarization modulation components lead to reduced accuracy in Stokes parameter inversion. Within the effective wavelength range, occasional minor exceedances of DoP over 1 originate from noise in the computational inversion process, with all such deviations being less than 5%.

Additionally, the vertical artifacts observed in the images of Stokes parameters $S_{2}$ and $S_{3}$ are associated with their weak signal characteristics (theoretical values of 0). Faint noise in measurements is amplified during the inversion process, resulting in stripes aligned with the push-broom direction of the spectrometer. This phenomenon is evident in both integrating sphere images measured under horizontal and vertical polarization directions.

### 3.2. Outdoor Polarization Measurement Experiment

#### 3.2.1. Experimental Setup and Measurement Protocols

An outdoor polarization imaging experiment was conducted to characterize the polarization properties of typical targets, including a car, rubber waterproof stickers, and military camouflage nets. The polarization imaging system (AHG-101 hyperspectral imager) was configured with an f/4 aperture, 15 ms exposure time, and 1 nm spectral resolution, ensuring consistent data acquisition across different targets and scenarios.

#### 3.2.2. Experimental Objects and Measurement Parameters

1. Car (Metallic Surface)

- Measurement time: 10:00 A.M.
- Geometric configuration: Vertical shooting from a rooftop at 50 m altitude toward a parking lot 200 m away
- Illumination conditions: Diffuse sunlight (solar elevation angle of 49.2° and azimuth angle of 123.4°)

2. Rubber Waterproof Stickers

- Measurement time: 10:15 A.M.
- Geometric configuration: Close-range shooting at 3 m distance with normal incidence
- Surface properties: Nonmetallic, diffuse reflection dominant

3. Military Camouflage Nets

- Group 1 (Sun-facing Shooting):

The first group of camouflage net measurements was carried out at 09:41 A.M., when the sun had an elevation angle of 45.5° and an azimuth angle of 118.4°. The imaging was conducted at an elevation angle of 11.5° toward the sun, with the imaging system directly facing the solar direction.

- Group 2 (Sun-back Shooting):

The second group of measurements on the camouflage net was performed at 3:10 P.M., with the sun at an elevation angle of 41.2° and an azimuth angle of 246.7°. In this scenario, the imaging system shot at an elevation angle of 11.5° away from the sun, capturing the target with the solar position behind the observation direction.

#### 3.2.3. Data Reconstruction and Results

On the basis of the raw polarization intensity data acquired by the imaging system (f/4 aperture, 15 ms exposure time, and 1 nm spectral resolution), full Stokes vector images ($S_{0},S_{1},S_{2},S_{3}$) and degree of polarization (DoP) images were reconstructed for all outdoor targets (cars, rubber waterproof stickers, and military camouflage nets). The main focus of the reconstruction process was preserving the details of spatial polarization features. The DoP images were calculated using Formula (12) to quantify the polarization intensity on the target surfaces, ensuring the integrity of the polarization features required for subsequent material discrimination.

(31) $$Dop=\frac{\sqrt{{S_{1}}^{2}+{S_{2}}^{2}+{S_{3}}^{2}}}{S_{0}}$$

**Figure 10:**
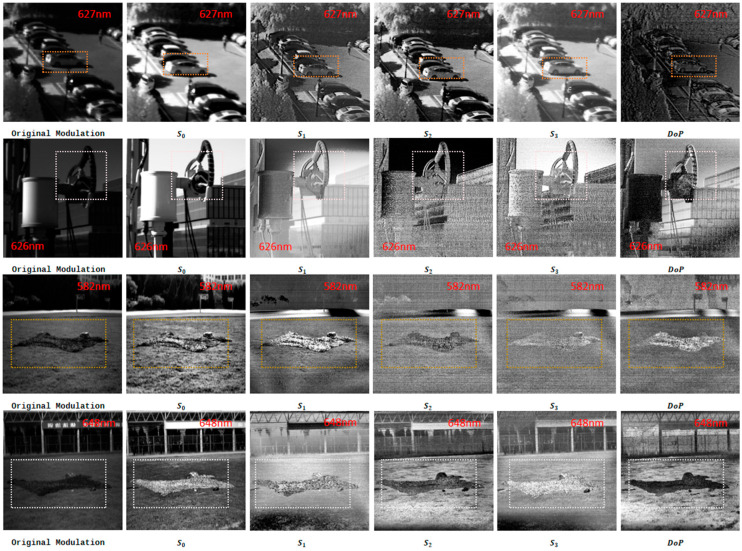
Original modulated images and reconstructed $S_{0}$, $S_{1}$, $S_{2}$, $S_{3}$, and DoP images under different environments. The first row shows the parking lot at a distance of 200 m; the second row shows the waterproof sticker at a distance of 3 m; and the third and fourth rows show the camouflage nets at different times and different solar angles.

**Figure 11:**
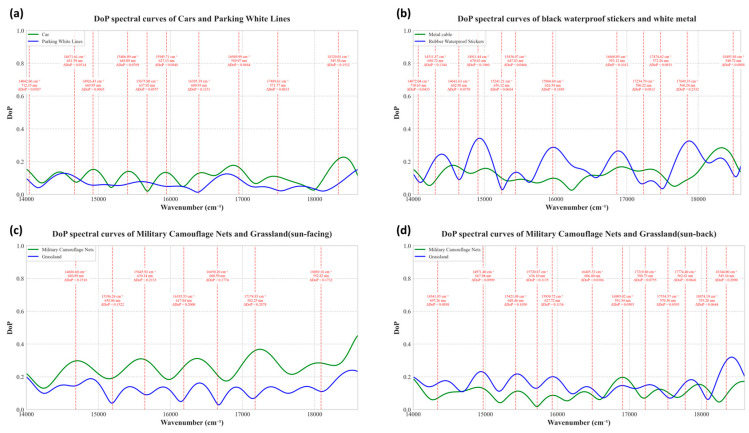
Comparative DoP spectral curves of typical outdoor targets under different scenarios: (**a**) vehicles and white parking lines in a parking lot; (**b**) black waterproof stickers and metal cables; (**c**) military camouflage nets & grassland (sun-facing, 09:41 A.M.); (**d**) military camouflage nets & grassland (sun-back, 3:10 P.M.). The red vertical dashed lines in all subfigures indicate the maximum difference points of the DoP between the two materials.

The Stokes vector components ($S_{0}$, $S_{1}$, $S_{2}$, and $S_{3}$) and degree of polarization (DoP) were reconstructed for all the targets. The imaging results (as shown in Figure 10) are compared with the DoP spectral curves of each material presented in Figure 11.

The reconstructed full Stokes vector images and DoP images effectively enhanced the discriminability of different materials, addressing the low-contrast issue in the original modulation images or $S_{0}$ intensity images. Combined with the experimental results presented in Figure 10 and Figure 11, the specific performance in each target scenario is detailed as follows.

1. Parking Lot Scene: Cars and Parking White Lines

In the reconstruction results of the parking lot scene (at 627 nm), the original modulation images and $S_{0}$ images could not highlight target details because of ambient light interference. In contrast, the use of the Stokes vector images and DoP images significantly improved material discrimination.

For cars, the $S_{1}$ and $S_{2}$ images clearly exhibited specular polarization features—bright and continuous polarization signals appeared on smooth metallic surfaces (e.g., car bodies and door panels). This phenomenon is attributed to the strong linear polarization effect generated by the specular reflection of sunlight on metal surfaces. In the visible-band DoP images, the cars showed distinct polarization intensities, creating a sharp contrast with the surrounding low-polarization environment (e.g., concrete ground), which made the contours and positions of the cars easy to distinguish.

For the parking white lines in shadows, the original modulation images and $S_{0}$ images showed poor visibility (blending with the dark ground under street lamp illumination). However, the Stokes vector images (especially the $S_{1}$ images) enhanced the difference in polarization between the white lines and the ground. The DoP images further amplified this contrast: the white lines appeared brighter against the dark ground, turning the originally indistinct white lines into clearly visible ones.

2. Rubber Waterproof Sticker Scene: Discrimination from Metal Cables

In the second group of experiments focused on rubber waterproof stickers, the original images failed to distinguish the black waterproof stickers from adjacent metal cables (both appeared dark and blended together). Nevertheless, the reconstructed Stokes vector images and DoP images (at 626 nm) resolved this ambiguity.

The Stokes vector images ($S_{1}$, $S_{2}$, $S_{3}$) of the waterproof stickers showed relatively high brightness, which reflects the weak but uniform polarization signals generated by diffuse reflection on the matte rubber surfaces. Despite this, the difference in brightness between the waterproof stickers and metal cables in the Stokes vector images was still insufficient for clear discrimination. DoP images effectively solved this problem: the rubber waterproof stickers appeared brighter because of their stable diffuse polarization characteristics, whereas the black metal cables showed lower brightness because of their strong specular reflection (which suppressed polarization uniformity). This obvious contrast in brightness directly distinguished the two black materials that were indistinguishable in the original images.

3. Military Camouflage Net Scene: Sun-Facing vs. Sun-Back Imaging

In the first set of experiments (sun-facing imaging at 09:41 A.M., with an elevation angle of 11.5° and a solar elevation angle of 45.5°), the original modulation images and $S_{0}$ images could not distinguish the camouflage nets from the grass (both appeared green with low contrast). In contrast, at 582 nm, the Stokes vector images (especially the $S_{2}$ images) highlighted the camouflage nets as brighter (due to the anisotropic polarization of their fibrous structures), whereas the grass appeared darker (resulting from isotropic diffuse reflection and weak polarization signals). The DoP images further intensified this contrast: the camouflage nets showed moderate polarization brightness, and the grass showed low polarization darkness, clearly separating the two.

In the second set of experiments (sun-back imaging at 15:10, a shooting elevation angle of 11.5°, and a solar elevation angle of 41.2°), the material discrimination effect was even more prominent. At 648 nm, the DoP images showed a reversed but clearer contrast: the camouflage nets appeared less bright (due to reduced polarization signals caused by backlighting), whereas the grass appeared brighter (due to enhanced diffuse polarization under backlighting). This distinct difference in brightness effectively resolved the issue of similar colors between the camouflage nets and the grass, achieving reliable material discrimination.

Furthermore, spectral analysis of the DoP curves for various materials (as shown in Figure 10) enables the identification of sensitive wavelength bands where the DoP differences between materials are maximized. This allows for the reduction of redundant band utilization in subsequent experiments—instead of processing images across all the bands, only the data from these optimal discriminative bands need to be used to obtain the most effective material-distinguishing images. Notably, the current study focused on a limited range of materials, as the primary objective herein is to propose a polarization processing framework. The systematic identification of sensitive bands for a broader spectrum of materials is reserved for future research endeavors.

## 4. Discussion

This study realized the miniaturized design of polarization imaging systems, enabling the acquisition of full Stokes vector data while ensuring spectral resolution and spatial resolution through the integrated design of a push-broom imaging spectrometer and modulator. Unlike traditional polarization imaging systems (such as early devices based on multisensor or mechanical rotating components [11,21]), the integrated design retains the capability of polarization feature extraction while reducing the system volume by 70% and shortening the single-scene acquisition time from minutes to seconds, enabling field deployment of portable polarization imaging devices.

Early polarization studies (e.g., [22]) have confirmed that polarization features can distinguish targets from similar-color backgrounds, but their application scenarios have long been limited to static laboratory environments or fixed-platform observations because of system volume and weight constraints (typical devices exceeding 5 kg). Through the modular push-broom design (spectrometer weight: 1.5 kg; modulator thickness: 55 mm), this study first validated the “palm-sized” polarization imaging system in dynamic outdoor scenarios. For example, in parking lot scenes, the device can acquire polarization images of cars and the ground in real time (acquisition time < 5 s), providing a key technological breakthrough for applications requiring high portability (such as individual reconnaissance and field environmental monitoring). This study verified the feasibility of polarization-based material discrimination in three typical outdoor scenarios: parking lots (cars vs. concrete, white lines vs. shadowed ground), dark material pairs (rubber waterproof stickers vs. metal cables), and similar-color camouflaged targets (camouflage nets vs. grass). The results show that Stokes vector images can capture surface polarization differences caused by distinct reflection mechanisms—for instance, metallic surfaces exhibit strong specular polarization (highlighted in $S_{1}$/$S_{2}$), whereas rubber materials show weak diffuse polarization (bright in all Stokes components). This observation aligns with the physical principle that polarization states are determined by material surface roughness and the refractive index [5,34], further validating that polarization features are inherent material attributes independent of spectral information. Notably, the reversed contrast between camouflage nets and grass in sun-facing/sun-back DoP images provides new insights for polarization imaging applications.

Despite the effective material discrimination performance, this study’s intensity modulation-based polarization imaging system has an inherent limitation: the spectral resolution of the Stokes vector components is constrained by the system’s sampling mechanism, which directly affects the accuracy of polarization feature extraction for targets with fine-scale surface structures.

As derived in Equation (32) of this study:

(32) $$∆\sigma_{max}=\frac{\sigma_{max}-\sigma_{min}}{S_{N}}\times \frac{1}{7}$$

Here, $\sigma_{max}$ represents the maximum wavenumber (in cm^−1^) of the spectral range under investigation, $\sigma_{min}$ denotes the minimum wavenumber, and $S_{N}$ is the number of sampling points in this range. The term $\frac{\sigma_{max}-\sigma_{min}}{S_{N}}$ corresponds to the original spectral sampling resolution of the system, whereas the factor $\frac{1}{7}$ arises from the intensity modulation mechanism—specifically, the multiplexing of spatial sampling points for the Stokes vector calculation leads to a 7-fold reduction in the effective spectral resolution of the Stokes components. This limitation is a fundamental trade-off of the intensity modulation principle—while the system enables synchronous polarization data acquisition, it inherently sacrifices spectral fineness compared with systems with dedicated polarization sensors (e.g., division-of-focal-plane polarimeters [25,30]). Unlike in DOFP systems, where resolution loss can be mitigated via nanofabricated micropolarizer arrays [32,33], the spectral resolution reduction in intensity modulation systems cannot be easily circumvented without redesigning the sampling mechanism.

In future work, we will conduct more refined measurements of circular polarization to further validate and improve the system’s performance in this regard. Additionally, in future research, due to the non-uniformity introduced by the slit and detector array, it will be necessary to perform flat-field correction on the polarimetric imaging system—specifically, conduct radiometric calibration to eliminate this effect. The portable polarization imaging system in this study is highly important for both academic research and practical applications. It advances the field of polarization imaging by demonstrating that portable, high-speed systems can achieve effective material discrimination in dynamic outdoor environments—an area previously dominated by bulky, static laboratory setups. The discovery of illumination angle-dependent polarization contrast also provides a new framework for interpreting polarization data in time-varying scenarios. Practically, the portability and real-time performance of the system make it a promising tool for military reconnaissance (e.g., camouflage detection), industrial defect inspection (e.g., dark material misalignment), and autonomous navigation (e.g., low-light parking assistance), where traditional spectral or visual imaging often fails.

To address the spectral resolution limitation of the intensity modulation system and further expand its capabilities, two key future research directions are proposed. First, integrating the intensity modulation framework with nanoscale micropolarizer arrays (e.g., UV-NIL-fabricated MPAs [32,33]) could reduce the 7-fold spectral resolution loss by minimizing spatial sampling multiplexing. Second, developing machine learning models (e.g., CNNs) to reconstruct high-resolution Stokes vector images from low-resolution raw data—building on the successes in DOFP polarimeter research [25]—could compensate for spectral fineness loss while retaining the portability of the system. Additionally, fusing polarization features with low-cost spectral sensors (e.g., 5–10 nm resolution modules) could enhance discrimination for materials with similar polarizations but distinct spectral responses, balancing accuracy and deployment feasibility for field applications.

## 5. Conclusions

Based on the polarization modulator and push-broom imaging spectrometer, we completed the design and verification of a sensor system for acquiring polarimetric spectral imaging information. Indoor and outdoor measurement experiments demonstrate that the sensor system achieves the portable design goals of small volume and light weight, enabling the simultaneous acquisition of polarimetric spectral imaging information. Its ability to obtain multi-dimensional information endows the sensor with potential application prospects in fields such as Earth environmental remote sensing, camouflaged target recognition, and astronomical observation.

## Figures and Tables

**Table 1:** Normalized Stokes Vector Components ($S_{1}$, $S_{2}$, and $S_{3}$) for Horizontal and Vertical Polarization at Different Wavelengths.

Polarization Direction	Wavelength (nm)	Wavenumber (cm^−1^)	$S_{1}$	$S_{2}$	$S_{3}$
0°	559.77	17,863.1	1.0059 ± 0.03	0.0101 ± 0.005	0.0274 ± 0.01
	639.76	15,631.0	0.9798 ± 0.02	0.0253 ± 0.01	0.0017 ± 0.005
	719.83	13,892.2	1.0212 ± 0.04	0.0165 ± 0.008	-0.0102 ± 0.02
90°	559.77	17,863.1	−0.9750 ± 0.03	−0.0287 ± 0.006	−0.0057 ± 0.015
	639.76	15,631.0	−0.9467 ± 0.02	−0.0109 ± 0.004	0.0070 ± 0.002
	719.83	13,892.2	−0.9773 ± 0.03	−0.0259 ± 0.007	0.0256 ± 0.01

Note: The values of $S_{1}$, $S_{2}$ and $S_{3}$ in the table are normalized results with $S_{0}$ set to 1, presented as the mean ±1σ (where 1σ is the standard deviation from 5 repeated measurements). The error calculation in Table 1 follows: relative error ($\left|\frac{S_{measured}-S_{theoretical}}{S_{theoretical}}\right|\times 100\%$) for non-zero theoretical values, and absolute error ($\left|S_{measured}-S_{theoretical}\right|\times 100\%$) for zero theoretical values. For each measurement, errors were computed for 10,000 pixels (100 × 100) near the center of the integrating sphere projection, with their mean taken as a single measurement result. The “average error” is the mean of 5 repeated measurements, and 1σ is the standard deviation of these 5 measurements. Theoretical values defined by an integrating sphere (Labsphere USLR-A12F-XAN2-P, Labsphere, North Sutton, NH, USA) and a linear polarizer (extinction ratio 10,000:1) on a THOR LABS PRM1 rotation stage (angular error ±0.1°). The theoretical Stokes vector for 0° polarization is $\left[S_{0},S_{1},S_{2},S_{3}\right]=[I,I,0,0]$, and that for 90° polarization is [s0,−s1,0,0] (where I is the total light intensity). Measurements were conducted using an AHG-101 hyperspectral imager (spectral resolution of 1 nm, spatial resolution of 960 × 960 pixels, and integration time of 5 ms).

## Data Availability

The raw data supporting the conclusions of this article will be made available by the authors on request.
